# Effectiveness of interventions for treating apophysitis in children and adolescents: protocol for a systematic review and network meta-analysis

**DOI:** 10.1186/s12998-018-0209-8

**Published:** 2018-10-23

**Authors:** Stig L. Midtiby, Niels Wedderkopp, Rasmus T. Larsen, Anne-Marie Fiala Carlsen, Dimitris Mavridis, Ian Shrier

**Affiliations:** 1 Department of Physiotherapy, University College Lillebaelt, Niels Bohrs Allé 1, 5230 Odense, SE Denmark; 2 Department of Regional Health Research, University of Southern, Copenhagen, Denmark; 3 Copenrehab, Section of Social Medicine, Department of Public Health, University of Copenhagen, Copenhagen, Denmark; 4 Department of Primary Education, University of Ioannina, Ioannina, Greece; 5 Centre for Clinical Epidemiology, Jewish General Hospital, McGill University, Ottawa, Canada; 6 Orthopedic department, Hospital of South Western Denmark, Esbjerg, Denmark

**Keywords:** Systematic review, Network meta-analysis, Child; overuse injury, Apophysitis, Osgood-Schlatter, Sinding-Larson-Johansson, Severs disease, Treatment

## Abstract

**Background:**

Overuse injuries are reported to be more common than acute trauma in children and adolescents, causing pain and reduced function. The most common is apophysitis - a traction injury to the apophysis in growing individuals. The duration of symptoms reported in the literature is between 6 weeks to 6 months or more. The objective of this systematic review and network meta-analysis is to compare the effectiveness and safety of all available treatments for any type of apophysitis in children and adolescents.

**Methods/Design:**

We will conduct a systematic review to retrieve all relevant studies applying a comparative design. Searches will be made in the Cochrane CENTRAL, MEDLINE, EMBASE, CINAHL and SportDiscus databases and via reference searching. The efficacy of treatments will be compared with respect to the outcomes 1) time to pain-free activity and 2) risk of subsequent injury. Risk of bias assessment will be made using *revised tool for assessing risk of bias in randomized trials* for Randomized trials and Robins-I tool for non-randomized trials. We will explore if different treatment comparisons are sufficiently similar in terms of effect modifiers (transitivity assumption) with the aim to conduct network meta-analyses for randomized and non-randomized studies separately. A treatment hierarchy will be obtained using the surface under the cumulative ranking curve (SUCRA) and mean ranks, visualized using rankograms. We will use the CINeMA software to apply the modified version of Grades of Recommendation, Assessment, Development and Evaluation (GRADE), developed specifically to evaluate the quality of evidence in network meta-analysis.

**Discussion:**

To date the comparative effects of interventions for apophysitis seem to rely mainly on expert opinion. We aim to identify all comparative treatment designs described in the literature and synthesize data when possible. We will use the estimated treatment effects between injury locations to provide guidance in managing apophysitis.

**Trial registration:**

PROSPERO ID number: CRD42018083746.

**Electronic supplementary material:**

The online version of this article (10.1186/s12998-018-0209-8) contains supplementary material, which is available to authorized users.

## Background

Overuse injuries are reported to be more common than acute trauma in children and adolescent, causing pain and reduced function [1]. In this population the most frequent overuse injuries are apophysitis, a traction injury to the apophysis with common names such as Osgood-Schlatters disease, Sever’s disease, and Pitchers elbow [2]. For example Osgood-Schlatters disease, an apophysitis at the patellar tendon’s attachment to the tibial tuberosity, has been reported to last more than 6 months [3], a period in which physical activity is restricted because of pain. Thus, the motivation for sports and physical activity may decrease, resulting in risk of obesity and later on various lifestyle diseases [4].

The apophysis consists of three chondrocyte zones: The resting germinal or stem cells represent the reserve zone, which is located adjacent to the apophysis. It consists of irregularly stacked chondrocytes and demonstrates a low rate of proliferation. This zone is critical because injury can result in growth cessation. The resting germinal cells enter the second proliferative zone. The proliferative zone consists of stacked columnar chondrocytes. These activated cells produce the extracellular matrix for longitudinal growth via active cell division. The chondrocytes hypertrophy and travel further from the apophysis into the third hypertrophic zone which is not involved in active growth; rather, it is responsible for maturation, degeneration, and provisional calcification. The hypertrophic zone is the weakest zone in the apophysis and is the site most often involved in alterations as widening and in the rare cases of avulsion fractures. Apophyseal stresses in overuse injuries have several potential sequelae including physeal widening, early calcification, avulsion fractures, and premature closure [5].

Injury to the vascularity of the apophysis can lead to disruption of the normal cycle of chondrocyte-programmed cell death and subsequent ossification. Radiographic findings are demineralization, fragmentation and apophyseal widening, and in some cases hypertrophy and bone prominence (e.g. tuberositas tibia in Osgood-Slater). These findings are usually not permanent and are expected to resolve with adjustment of activity, except for the bony prominence [6].

In a recent clinical overview by Arnold and colleagues [2], the interventions for apophysitis are discussed, but no attempt on quantitative data synthesis has been made.

The objective of this systematic review and network meta-analysis is to compare the effectiveness and safety of all available treatments for any type of apophysitis in children and adolescent. We will compare the efficacy of treatments with respect to the outcomes 1) time to pain-free activity and 2) risk of subsequent injury. We will follow the Preferred Reporting Items for Systematic Reviews and Meta-Analyses (PRISMA) guidelines for Network Meta-Analyses [7].

## Methods

### Criteria for considering studies for this review

#### Types of studies

Our initial strategy will be to include all studies that provide longitudinal data from a comparative design on any of our primary or secondary outcomes. As we do not expect many randomized controlled trials we will include non-randomized comparative designs such as prospective cohort studies (including nested case-control analyses), historical cohort studies (including nested case-control analyses) and Non-nested case-control study design.

#### Types of participants

Participants must be 17 years of age or younger at the time of diagnosis. We will include studies involving both sexes, with apophysitis at any location.

#### Types of interventions

We will include studies examining any intervention for an apophysitis at any location. We are aware that the following treatments could currently be used by clinicians in clinical practice: usual care (wait and see), specific exercises, reduction of specific physical activity, taping, bracing, manual therapy, joint or tissue mobilization, dry needling, cryotherapy, electrical modalities, medication and surgery. The interventions are further described in ‘Additional file 1 description of interventions’.

#### Types of outcome measures

Our primary outcome will be the duration of treatment from start of treatment until return to previous activity level without pain. If this is not provided in a particular study, we will still consider return to previous activity with or without pain as an equivalent outcome. Our secondary outcomes include: Time from treatment initiation to return to sport, risk of any subsequent injury (any location, any type - including acute and overuse injuries and risk of subsequent injury to the same location of the same type) and risk of recurrent injury (same location and same type) [8]. We define a subsequent or recurrent injury as a musculoskeletal injury as a result physical activity that requires treatment from a clinician.

### Search methods for identification of studies

We will search the following databases: Cochrane CENTRAL, MEDLINE, EMBASE, CINAHL and SportDiscus. We will search the references of all retrieved articles and conduct a citation search of key articles retrieved from our primary searches for potentially relevant titles. Papers written in English, Danish, Swedish, Norwegian and German will be included.

#### Search strategy

Our search strategy combines three constructs: treatment/rehabilitation, apophysitis, and children/adolescents. ‘Additional file 2 search strategy’ describes our currently planned search strategy. If this strategy appears to identify many non-applicable types of articles, we will adapt the strategy to restrict its focus. Similarly, the search strategy will be adapted if the reference search provides many relevant studies, undetected by the original strategy.

### Data collection and analysis

#### Selection of studies

The same two people (SLM, RTL) will review the titles and abstracts to select eligible studies; discrepancies at any stage will be resolved via consensus and will include the remaining co-investigators of our study if necessary. The reviewers will independently read all titles retrieved from the search strategy to exclude any obviously non-relevant papers. SLM and RTL will then review all abstracts where titles were included by one of the abstractors, and document the reasons why a particular study is excluded. The reviewers will then review the full articles, considered relevant by one of the abstractors, and document the reasons why if the paper is excluded at this stage.

We will extract the following study level characteristics: lead author, year of publication, journal, setting (e.g. hospital, private clinic), intervention details (such as medication, exercise, manual therapy and surgery) and outcome measures. We will extract data on participant level characteristics: age, gender, onset of puberty (yes/no), sport/physical activity, time to treatment, injury location, severity of injury, time to return to activity, time to return to pain-free activity, subsequent injury risk for any injury or recurrent injury (see outcomes) and number of dropouts including reason for dropout (if mentioned). Data extraction will be carried out independently by SLM and RTL using standard data extraction forms. Data will be cross checked between reviewers. The extracted data will be discussed in case of discrepancy between the reviewers and differences will be resolved by the same consensus process as above.

#### Data on potential effect modifiers

Clinical features we believe may affect efficacy of treatments are: age, gender, onset of puberty (defined by Tanner stage [9]), sport/physical activity, time to treatment, treatment dosage, if exercises are primarily supervised or homebased, injury location, severity and study type. The effects of these features may differ between injury locations. We will collect data on all of these variables as noted in the preceding section.

#### Assessment of risk of bias in included studies

For randomized controlled trials, we will assess the risk of bias with the *revised tool for assessing risk of bias in randomized trials* [10]. Two independent reviewers will assess all domains including bias arising from randomization process, deviations from intended interventions, missing outcome data, measurement of the outcome, selection of the reported result and other biases particular to special study designs. Each risk of bias item will receive either “low”, “some concerns” or “high” risk of bias, and each judgement will include a support statement as recommended by the tool. Discrepancies will be resolved by consensus and will include the remaining co-investigators of our study if necessary.

For non-randomized studies, we will use the ROBINS-I tool [11] to assess the risk of bias in each of the following seven domains: Bias due to confounding, selection of participants into the study, classification of interventions, deviations from intended interventions, missing data, measurement of outcomes and selection of the reported result. The categories for risk of bias judgements are “Low risk”, “Moderate risk”, “Serious risk” and “Critical risk” of bias. Importantly, “Low risk” corresponds to the risk of bias in a high quality randomized trial [11]. The assessment will be performed independently by two raters and discrepancies will be resolved via consensus and will include the remaining co-investigators of our study if necessary.

### Measures of treatment effect

#### Relative treatment effects

The main outcome: days until pain-free activity will be expressed as mean differences. We will calculate the risk of subsequent injury (over the time periods provided in the original studies) for both treatment groups. For dichotomous outcomes, we will report risk ratios, risk differences and number needed to treat. For continuous outcomes, we will report mean differences unless the outcome is measured differently across studies in which case we will report standardized mean differences. We will perform a network meta-analysis [12, 13], which is a method that synthesizes direct and indirect evidence and allows us to compare the relative effectiveness between treatments that have never been compared head-to-head. Results from network meta-analysis will be presented as summary relative effect sizes (mean differences or odds ratios (OR)) for each possible pair of interventions. We will produce summary results for all outcomes and give the respective 95% confidence intervals (95% CI). We will use restricted maximum likelihood to estimate heterogeneity assuming that heterogeneity is common in all treatment comparisons. If we have skewed data, we will use the methods presented by Higgins et al. to pool results [14].

#### Relative treatment ranking

We will estimate the ranking probabilities for all treatments of being at each possible rank for each intervention. Then, we will obtain a treatment hierarchy using the surface under the cumulative ranking curve (SUCRA) and mean ranks, visualized using rankograms [15]. The SUCRA statistics will be interpreted with reference to the corresponding relative effects and 95% CI between two treatments. This will also ensure that differences are clearly visualized and their clinical importance is taken into account.

### Unit of analysis issues

We will extract summary data but our unit of analysis is the individual participant. If there are any cross over studies, we will use both periods if the study provides evidence that there was no carry-over effect in their study design, and use only the first period if this evidence was not provided. Results from cluster randomized trials will be included with individual participant as unit of analysis. In case of errors in the studies, we will recalculate the standard errors on the effect size where possible.

### Dealing with missing data

We will report missing data and reasons for dropout in all included studies. Whenever there are missing data in the original papers, we will contact the authors to obtain whatever information is possible. If we deem that reasons for dropout are related to the outcomes, we will consider the study to be at high risk of bias. If studies have used single imputation techniques (such as last observation carried forward, mean imputation etc.) we will consider the study to be at high risk of bias. In such cases, we will conduct a sensitivity analysis using a pattern mixture model [16, 17] to evaluate how robust results are to departures from the missing at random assumption.

### Assessment of clinical and methodological heterogeneity within treatment comparisons

We will assess the presence of clinical heterogeneity within each pairwise comparison by describing the trial and study population characteristics across all eligible trials.

### Assessment of transitivity across treatment comparisons

The transitivity assumption, which is the underlying assumption for network meta-analysis, will be assessed by comparing the distribution of potential effect modifiers across each different pairwise comparison. If a common comparator in a Network differs from one pair-wise comparison to another the transitivity assumption is violated and the validity of the network meta-analysis results is compromised [12].

### Data synthesis

We will first analyze randomized studies and observational studies separately. For observational studies, we will use adjusted measures when provided and appropriate.

### Methods for direct treatment comparisons

First, we will conduct pair-wise meta-analyses by synthesizing studies that compare interventions, regardless of injury location. The same pairwise meta-analyses will be performed in interventions for a specific injury location (e.g. knee, heel or elbow) using a random-effects model if sufficient studies are available. All statistics will be conducted in STATA (StataCorp. 2011. Statistical software: Release 14. College Station, TX).

### Methods for indirect and mixed comparisons

We assume that any patient that meets the inclusion criteria is, in principle, equally likely to be randomized to any of the eligible interventions. We hope to conduct a network meta-analysis provided we are able to collect sufficient data. If not, we will conduct standard meta-analyses with meta-regression (where appropriate) for particular treatment comparisons both overall and separate anatomic locations (e.g. knee, heel or elbow), and a qualitative evidence synthesis where meta-analyses are not appropriate. We will perform network meta-analysis using STATA (StataCorp. 2011. Statistical software: Release 14. College Station, TX) using the network package [18] and self-programmed STATA routines [19] available at http://mtm.uoi.gr.

### Assessment of statistical heterogeneity

We will explore whether treatment effects for our primary outcomes are robust in subgroup analyses and network meta-regression using the following characteristics: Age, gender, onset of puberty, sport/physical activity, time to treatment, treatment dosage, if exercises are primarily supervised or homebased, injury location, severity, and study type. We will analyze each characteristic separately and finally include in a meta-regression those characteristics that were found to be statistically significant in the univariate analyses.

#### Assumptions when estimating the heterogeneity

In standard pairwise meta-analyses we will estimate different heterogeneity variances for each pairwise comparison within a random effects model framework.

#### Measures and tests for heterogeneity

We will assess the presence of heterogeneity within each pairwise comparison using the I^2^ (percentage of variability that is due to differences in the underlying effects across studies) and τ ^2^ statistics.

The assessment of statistical heterogeneity in the entire network will be based on the magnitude of τ^2^estimated from the network meta-analysis models. For dichotomous outcomes the magnitude of the heterogeneity variance will be compared with the empirical distribution [20]. We will also estimate a total I^2^ value for heterogeneity in the network as described elsewhere.

### Assessment of statistical inconsistency

#### Local approaches for evaluating inconsistency

To evaluate the presence of inconsistency locally we will use the loop-specific approach. This method evaluates the consistency assumption in each closed loop of the network separately as the difference between direct and indirect estimates for a specific comparison in the loop (inconsistency factor). Then, the magnitude of the inconsistency factors and their 95% CIs can be used to infer about the presence of inconsistency in each loop. We will assume a common heterogeneity estimate within each loop. We will present the results of this approach graphically in a forest plot using the *ifplot* command in STATA.

#### Global approaches for evaluating inconsistency

To check the assumption of consistency in the entire network we will use the ‘design-by-treatment’ model as described by Higgins and colleagues [21].

### Investigation of heterogeneity and inconsistency

If we find important heterogeneity or/and inconsistency, we will explore the possible sources. If sufficient studies are available, we will perform meta-regression or subgroup analyses by using the following effect modifiers as possible sources of inconsistency and or heterogeneity: age, gender, onset of puberty, sport/physical activity, time to treatment, injury location, severity and study type.

#### Sensitivity analysis

Our primary analysis will include all studies retrieved through the search strategy described above. In sensitivity analyses, we will exclude [1] studies not reporting SD rather than imputed, [2] studies at high or unclear risk of bias and [3] outlying studies.

### Quality of evidence

We will use a modified version [22] of Grades of Recommendation, Assessment, Development and Evaluation (GRADE), developed specifically to evaluate the quality of evidence in network meta-analysis. For confidence in specific pairwise effect and treatment rankings estimated in the network meta-analysis the following domains will be evaluated: Study limitations, indirectness, inconsistency, imprecision, and publication bias. The starting point for confidence in each network estimate is high, but will be downgraded according to the assessments of these five domains. The GRADE process will be completed using the CINeMA software, developed by the Cochrane Statistics Methods Group for evaluating confidence in the results of network meta-analysis [22, 23].

## Discussion

Despite the high incidence of overuse injuries in children and adolescent [24] the comparative treatment effects for apophysitis seems to rely mainly on expert opinions [2]. Therefore, the objective of this systematic review and network meta-analysis is to compare the effectiveness and safety of all available treatments for any type of apophysitis in children and adolescent.

To identify all comparative treatment designs described in the literature, we chose a broad search strategy. We prefer to restrict the studies to high quality randomized controlled trials. However, as we do not expect many of these, we will include other comparative designs such as different types of cohort studies and case control studies. We will prioritize studies of high methodological quality. This strategy will able us to collect the most relevant research within the area of apophysitis.

Compared to the recent review by Arnold et al. [2] on the same topic, the current study implements a much broader search strategy as described in Additional file 2, and our strategy for data synthesis, including a network analysis, that will enable us to compare interventions if they use a common comparator. Further we plan to update our review using the present study protocol every 3rd year if more relevant research has been published, which will keep researchers and clinicians updated on the subject.

Our main outcome is time from treatment initiation to previous activity level without pain, which we believe is the most relevant outcome for patients, clinicians, trainers and etcetera. We expect to identify common patterns of treatment effects between injury locations, in order to guide clinicians in managing apophysitis.

A possible limitation to our study is the current quality of the evidence that appears to be generally low according to Arnold et al. [2]. Thus we might end up with very few studies for our network analysis.

## Additional files

Additional file 1: Description of interventions. (DOCX 16 kb)

Additional file 2: Search strategy. (DOCX 26 kb)
