# Supplementary material for: Effectiveness of interventions for treating apophysitis in children and adolescents: protocol for a systematic review and network meta-analysis
Source: Chiropr Man Therap. 2018 Oct 23;26:41. doi: 10.1186/s12998-018-0209-8 (PMC6198434; doi:10.1186/s12998-018-0209-8)
Supplement: Supplementary file 1 — Description of interventions. (DOCX 16 kb) [file 12998_2018_209_MOESM1_ESM.docx]

# Apendix 1: Further description of the interventions

Exercise interventions including: specific exercise interventions, to address functional deficiencies. Training load management: Reduction of specific sports or physical activities, in order to reduce symptoms.

Taping and bracing

- Some types of taping and bracing perform similar functions, however they are structurally different. Therefore the two interventions will be treated as separate in our primary approach. As a secondary approach taping and bracing interventions will be investigated separately. Bracing: any external device that reduces load on the injured tissue
- Taping: to reduce load or improve proprioception.
- Cryotherapy: includes ice and heat.
- The above can all be performed in different doses and as either supervised treatment or as homebased treatments after instruction. We will include the dose and whether an exercise treatment is constantly supervised or if it is primarily homebased and unsupervised in the analyzes.

Manual therapy: includes massage, mobilizations, high velocity manipulations

Dry needling, such as acupuncture.

Electrical modalities: includes modalities such as ultrasound, TENS, iontophoresis without medication, short wave diathermy, shock wave therapy and laser therapy.

Medication: includes analgesics, anti-inflammatories, cortisone, ibuprofen, naproxen, paracetamol, diclofenac, acetaminophen,

Surgery: Any surgical procedure designed for treatment of the condition.
