# Supplementary material for: Effectiveness of interventions for treating apophysitis in children and adolescents: protocol for a systematic review and network meta-analysis
Source: Chiropr Man Therap. 2018 Oct 23;26:41. doi: 10.1186/s12998-018-0209-8 (PMC6198434; doi:10.1186/s12998-018-0209-8)
Supplement: Supplementary file 2 — Search strategy. (DOCX 26 kb) [file 12998_2018_209_MOESM2_ESM.docx]

# Appendix 2. Search strategy

To identify the relevant literature the databases Cinahl Complete, Embase, SprotDISCUS, Medline via PubMed, and Cochrane Library were systematically searched. Through an analysis of text words in the first search, and through the searching, new keywords, which would improve the search, were identified. All identified keywords were then included in the search as a systematic block search.

## Search matrix:

| Block 1 | Block 2 | Block 3 |
| --- | --- | --- |
| child | physis injury/ies | taping |
| children | physeal injury/ies | tape |
| Adolescents | Osteochondrosis | acetaminophen |
| Adolescence | Thrower’s elbow | diclofenac |
| youth | sinding-larsen | paracetamol |
|  | growth plate | naproxen |
|  | Pitcher’s elbow | ibuprofen |
|  | apophysitis | anti-inflammatory |
|  | Osgood Schlatter | cortisone |
|  | sever's | ultrasound therapy |
|  | overuse injury/ies | ultrasonic therapy |
|  |  | laser therapy |
|  |  | acupuncture |
|  |  | dry needling |
|  |  | cryotherapy |
|  |  | high velocity thrust |
|  |  | high velocity manipulation |
|  |  | mobilization |
|  |  | massage |
|  |  | shock wave |
|  |  | short wave diathermy |
|  |  | manual therapy |
|  |  | physical therapy |
|  |  | physiotherapy |
|  |  | analgesic |
|  |  | treatment |
|  |  | therapy |
|  |  | Therapeutics |
|  |  | Rehabilitation |

| # | Final search  Cinahl Complete  November 6th, 2017 | Hits |
| --- | --- | --- |
| Blok 1 | children OR adolescents OR adolescence OR youth OR teenagers OR young adults | 867210 |
| Blok 2 | physis injuries OR physeal injuries OR growth plate OR Osteochondrosis OR thrower's elbow OR sinding-larsen OR apophysitis OR Osgood Schlatter OR sever's OR Pitcher’s elbow OR overuse injuries | 2208 |
| Blok 3 | taping OR tape OR acetaminophen OR diclofenac OR paracetamol OR ibuprofen OR naproxen OR anti-inflammatory OR cortisone OR ultrasound therapy OR ultrasonic therapy OR laser therapy OR acupuncture OR dry needling OR cryotherapy OR high velocity thrust OR high velocity manipulation OR mobilization OR massage OR shock wave OR short wave diathermy OR manual therapy OR physical therapy OR physiotherapy OR analgesia OR insole OR treatment OR therapy OR Therapeutics OR Rehabilitation | 1658668 |
| 1 AND 2 AND 3 | ( children OR adolescents OR adolescence OR youth OR teenagers OR young adults ) AND ( physis injuries OR physeal injuries OR growth plate OR Osteochondrosis OR thrower's elbow OR sinding-larsen OR apophysitis OR Osgood Schlatter OR sever's OR Pitcher’s elbow OR overuse injuries ) AND ( taping OR tape OR acetaminophen OR diclofenac OR paracetamol OR ibuprofen OR naproxen OR anti-inflammatory OR cortisone OR ultrasound therapy OR ultrasonic therapy OR laser therapy OR acupuncture OR dry needling OR cryotherapy OR high velocity thrust OR high velocity manipulation OR mobilization OR massage OR shock wave OR short wave diathermy OR manual therapy OR physical therapy OR physiotherapy OR analgesia OR insole OR treatment OR therapy OR Therapeutics OR Rehabilitation ) | 464 |

| # | Final search  Embase  November 6th, 2017 | Hits |
| --- | --- | --- |
| Blok 1 | child/ or children.mp. or Adolescent/ or Adolescence/ or Youth.mp. or juvenile/ | 2893588 |
| Blok 2 | (physis injury or physis injuries).mp. or epiphysis injury/ or physeal injuries.mp. or physeal injuries.mp. or epiphysis plate/ or growth plate.mp. or osteochondrosis/ or (thrower's elbow or sinding-larsen or Osgood Schlatter or Apophysitis or sever's or overuse injuries or overuse injury).mp. | 14393 |
| Blok 3 | Taping.mp. OR Tape.mp. OR Acetaminophen.mp. OR Diclofenac/ OR Paracetamol/ OR Naproxen/ OR Ibuprofen/ OR anti-inflammatory.mp. OR Cortisone/ OR ultrasound therapy/ OR ultrasonic therapy.mp. OR laser therapy.mp. OR Acupuncture/ OR dry needling.mp. OR Cryotherapy/ OR high velocity thrust OR high velocity manipulation OR high velocity thrust manipulation OR Mobilization/ OR Massage/ OR shock wave/ OR short wave diathermy/ OR manual therapy.mp. OR physical therapy.mp. OR Physiotherapy/ OR analgesic agent/ OR conservative treatment/ OR Therapy/ OR Therapeutics.mp. OR Rehabilitation/ | 2156317 |
| 1 AND 2 AND 3 | ( child/ or children.mp. or Adolescent/ or Adolescence/ or Youth.mp. or juvenile/ ) AND ( (physis injury or physis injuries).mp. or epiphysis injury/ or physeal injuries.mp. or physeal injuries.mp. or epiphysis plate/ or growth plate.mp. or osteochondrosis/ or (thrower's elbow or sinding-larsen or Osgood Schlatter or Apophysitis or sever's or overuse injuries or overuse injury).mp. ) AND ( Taping.mp. OR Tape.mp. OR Acetaminophen.mp. OR Diclofenac/ OR Paracetamol/ OR Naproxen/ OR Ibuprofen/ OR anti-inflammatory.mp. OR Cortisone/ OR ultrasound therapy/ OR ultrasonic therapy.mp. OR laser therapy.mp. OR Acupuncture/ OR dry needling.mp. OR Cryotherapy/ OR high velocity thrust OR high velocity manipulation OR high velocity thrust manipulation OR Mobilization/ OR Massage/ OR shock wave/ OR short wave diathermy/ OR manual therapy.mp. OR physical therapy.mp. OR Physiotherapy/ OR analgesic agent/ OR conservative treatment/ OR Therapy/ OR Therapeutics.mp. OR Rehabilitation/ ) | 483 |

| # | Final search  SportDISCUS  November 7th, 2017 | Hits |
| --- | --- | --- |
| Blok 1 | children OR Adolescents OR Adolescence OR youth OR Teenagers OR Young adults | 160073 |
| Blok 2 | physis injuries OR physeal injuries OR growth plate OR Osteochondrosis OR throwers elbow OR sinding-larsen OR Apophysitis OR Osgood Schlatter OR sever's OR Pitchers elbow OR overuse injuries | 4498 |
| Blok 3 | ( ( taping OR tape OR Acetaminophen ) OR diclofenac OR paracetamol OR naproxen OR ibuprofen OR anti-inflammatory OR cortisone OR ultrasound therapy OR ultrasonic therapy OR laser therapy OR acupuncture OR dry needling ) OR ( ( insole OR cryotherapy OR high velocity thrust ) OR ( high velocity manipulation OR mobilization ) OR ( massage OR shock wave OR short wave diathermy ) OR manual therapy OR ( physical therapy OR physiotherapy ) OR analgesia OR treatment OR therapy OR Therapeutics OR Rehabilitation ) | 238961 |
| 1 AND 2 AND 3 | ( physis injuries OR physeal injuries OR growth plate OR Osteochondrosis OR throwers elbow OR sinding-larsen OR apophysitis OR Osgood Schlatter OR sever's OR Pitchers elbow OR overuse injuries ) AND ( children OR Adolescents OR Adolescence OR youth OR Teenagers OR Young adults ) AND ( ( ( taping OR tape OR Acetaminophen ) OR diclofenac OR paracetamol OR naproxen OR ibuprofen OR anti-inflammatory OR cortisone OR ultrasound therapy OR ultrasonic therapy OR laser therapy OR acupuncture OR dry needling ) OR ( ( insole OR cryotherapy OR high velocity thrust ) OR ( high velocity manipulation OR mobilization ) OR ( massage OR shock wave OR short wave diathermy ) OR manual therapy OR ( physical therapy OR physiotherapy ) OR analgesia OR treatment OR therapy OR Therapeutics OR Rehabilitation ) ) | 521 |

| # | Final search  MEDLINE via PubMed  November 6th, 2017 | Hits |
| --- | --- | --- |
| Blok 1 | children OR Adolescents OR adolescence OR youth | 3194011 |
| Blok 2 | physis injury OR Physis injuries OR physeal injury OR physeal injuries OR (“growth plate"[MeSH Terms]) OR Osteochondrosis OR throwers elbow OR thrower's elbow OR “thrower's elbow” OR sinding-larsen OR Osgood Schlatter OR Apophysitis OR sever's OR “overuse injuries” OR "overuse injury" OR pitcher's elbow | 11177 |
| Blok 3 | (((taping OR tape OR acetaminophen OR diclofenac OR paracetamol OR naproxen OR ibuprofen OR anti-inflammatory OR cortisone OR "ultrasound therapy" OR ultrasonic therapy OR laser therapy OR acupuncture OR "dry needling" OR cryotherapy OR "high velocity thrust" OR "high velocity manipulation" OR massage OR "shock wave" OR "short wave diathermy" OR "manual therapy" OR "physical therapy" OR physiotherapy OR analgesic))) OR ((Rehabilitation[Title/Abstract] OR mobilization[Title/Abstract] OR mobilisation[Title/Abstract] OR treatment[Title/Abstract] OR therapy[Title/Abstract] OR Therapeutics[Title/Abstract])) | 5507745 |
| 1 AND 2 AND 3 | ((((((taping OR tape OR acetaminophen OR diclofenac OR paracetamol OR naproxen OR ibuprofen OR anti-inflammatory OR cortisone OR "ultrasound therapy" OR ultrasonic therapy OR laser therapy OR acupuncture OR "dry needling" OR cryotherapy OR "high velocity thrust" OR "high velocity manipulation" OR massage OR "shock wave" OR "short wave diathermy" OR "manual therapy" OR "physical therapy" OR physiotherapy OR analgesic))) OR (((Rehabilitation[Title/Abstract] OR mobilization[Title/Abstract] OR mobilisation[Title/Abstract] OR treatment[Title/Abstract] OR therapy[Title/Abstract] OR Therapeutics[Title/Abstract]))))) AND ((physis injury OR Physis injuries OR physeal injury OR physeal injuries OR (“growth plate"[MeSH Terms]) OR Osteochondrosis OR throwers elbow OR thrower's elbow OR “thrower's elbow” OR sinding-larsen OR Osgood Schlatter OR Apophysitis OR sever's OR “overuse injuries” OR "overuse injury" OR pitcher's elbow))) AND ((children OR Adolescents OR adolescence OR youth)) | 1596 |

| # | Final search  Cochrane library  November 6th, 2017 | Hits |
| --- | --- | --- |
| Blok 1 | children or Child or Adolescents or adolescence or youth | 126336 |
| Blok 2 | physis injury or Physis injuries or physeal injury or physeal injuries or growth plate or Osteochondrosis or throwers elbow or thrower's elbow or sinding-larsen or Osgood Schlatter or Apophysitis or sever's or overuse injuries or overuse injury or pitcher's elbow | 467 |
| 1 AND 2 | #1 and #2 | 158 |

Records identified through database searching

(n= 3222)

Embase (n = 483)

Cinahl (n= 464)

PubMed (n= 1596)

SportDiscus (n= 521)

Cochrane library (n= 158)

Records after duplicates removed
(n = 2708)

Dubletter (n = 514):

Embase = 8

Cinahl = 57

SportDiscus = 108

PubMed = 320

Cochrane = 21
